# Perfusion Imaging of Fatigue and Time-on-Task Effects in Patients With Parkinson’s Disease

**DOI:** 10.3389/fnagi.2022.901203

**Published:** 2022-06-10

**Authors:** Wanting Liu, Jianghong Liu, Rupal Bhavsar, Tianxin Mao, Eugenia Mamikonyan, David Raizen, John A. Detre, Daniel Weintraub, Hengyi Rao

**Affiliations:** ^1^School of Psychology, South China Normal University, Guangzhou, China; ^2^Center for Magnetic Resonance Imaging Research and Key Laboratory of Applied Brain and Cognitive Sciences, School of Business and Management, Shanghai International Studies University, Shanghai, China; ^3^Center for Functional Neuroimaging, Department of Neurology, University of Pennsylvania, Philadelphia, PA, United States; ^4^Department of Family and Community Health, University of Pennsylvania School of Nursing, Philadelphia, PA, United States; ^5^Department of Psychiatry, University of Pennsylvania Perelman School of Medicine, Philadelphia, PA, United States

**Keywords:** psychomotor vigilance test, Parkinson’s disease, fatigue, time-on-task effect, ASL perfusion fMRI, frontoparietal network

## Abstract

Fatigue is a highly prevalent and debilitating non-motor symptom in Parkinson’s disease (PD), yet its’ neural mechanisms remain poorly understood. Here we combined arterial spin labeling (ASL) perfusion functional magnetic resonance imaging (fMRI) with a sustained mental workload paradigm to examine the neural correlates of fatigue and time-on-task effects in PD patients. Twenty-one PD patients were scanned at rest and during continuous performance of a 20-min psychomotor vigilance test (PVT). Time-on-task effects were measured by the reaction time changes during the PVT and by self-reported fatigue ratings before and after the PVT. PD subjects demonstrated significant time-on-task effects, including progressively slower reaction time on the PVT and increased post-PVT fatigue ratings compared to pre-PVT. Higher levels of general fatigue were associated with larger increases in mental fatigue ratings after the PVT. ASL imaging data showed increased CBF in the right middle frontal gyrus (MFG), bilateral occipital cortex, and right cerebellum during the PVT compared to rest, and decreased CBF in the right MFG at post-task rest compared to pre-task rest. The magnitude of regional CBF changes in the right MFG and right inferior parietal lobe correlated with subjective fatigue rating increases after the PVT task. These results demonstrate the utility of continuous PVT paradigm for future studies of fatigue and cognitive fatigability in patients, and support the key role of the fronto-parietal attention network in mediating fatigue in PD.

## Introduction

Parkinson’s disease (PD) is the second most common neurodegenerative disease, affecting millions of people worldwide. PD patients typically show a resting tremor, rigidity, bradykinesia, and postural instability (Hoehn and Yahr, 1967; Tandberg et al., 1995). While these motor signs are considered cardinal features of PD, there is mounting evidence that PD is a systemic disease that also includes non-motor signs and symptoms (Hoehn and Yahr, 1967; Friedman and Friedman, 1993; Pfeiffe, 2016). Of these non-motor symptoms, fatigue, both mental and physical, is one of the most common and disabling (Pfeiffe, 2016). Among PD patients, the prevalence of fatigue is 33–70% (Friedman and Friedman, 1993; van Hilten et al., 1993; Friedman et al., 2011; Herlofson et al., 2012), and it may even precede the onset of motor symptoms (Hagell and Brundin, 2009). About one-third of PD patients report that fatigue is the most disabling symptom and significantly impairs their daily activities and reduces their overall quality of life (Herlofson and Larsen, 2003; Friedman et al., 2007; Kluger et al., 2014). However, fatigue tends to be under-diagnosed, and there are no effective treatments. Currently, in clinical settings, fatigue assessments rely largely on subjective patient self-reports, but there is a growing interest in objective assessments of fatigue in PD for earlier identification of physical indicators of fatigue. Furthermore, understanding of the neural mechanisms underlying fatigue in PD could potentially provide significant insights for developing prevention and management strategies.

Fatigue includes both physical and mental/cognitive manifestations. However, progress in PD fatigue research is partly hinded by issues of definition and measurement of fatigue, particularly its cognitive aspects. Kluger et al. suggested distinguishing subjective sensations of exhaustion (i.e., perceived fatigue) from objective decrement in cognitive performance or physiological capacity induced by continued activity (i.e., fatigability) (Kluger et al., 2013). Cognitive fatigability is often measured as declines in either reaction time (RT) or accuracy over time on continuous cognitive tests. Perceived fatigue experience and fatigability may be dissociable. For example, although PD patients demonstrate heightened fatigability on both cognitive and motor tasks, such fatigability does not correlate with subjective fatigue complaints (Lou, 2009). This highlights the importance of considering both objective and subjective measurements of fatigue in PD patients. More studies are needed to establish the relationship between these two components. It is possible that the appropriate choice of performance metric may improve our ability to examine the associations between self-reported fatigue and objectively measured cognitive fatigability (Wang et al., 2014).

Several studies have examined the neural bases of subjective fatigue complaints in PD. For example, reduced perfusion in the prefrontal cortex and decreased serotonergic activity in the basal ganglia and limbic regions has been reported in fatigued PD patients (Abe et al., 2000; Pavese et al., 2010). PD patients with fatigue also showed abnormal metabolic changes in the in the salience network and default mode network (DMN) regions (Cho et al., 2017). Using blood oxygenation level dependent (BOLD) functional magnetic resonance imaging (fMRI), Tessitore et al. (2016) found that primary PD-related fatigue is associated with increased connectivity within the prefrontal and posterior cingulate hubs of the DMN in drug-naive patients (Tessitore et al., 2016). These findings are consistent with the model of central/cognitive fatigue proposed by Chaudhuri and Behan (2000), which hypothesizes that central fatigue is associated with impairment of basal ganglia non-motor functions and negatively impacts the striatal-thalamic-frontal cortical system.

In contrast to subjective patient fatigue studies, few if any studies examined the neural correlates of objective cognitive fatigability in PD patients. To the best of our knowledge, there are no reported brain imaging studies that have probed the neural underpinnings of cognitive fatigability in PD from mentally demanding tasks. Early imaging studies on fatigue typically employed positron emission tomography (PET) or BOLD fMRI (Tessitore et al., 2016; Cho et al., 2017). However, PET has poor temporal and spatial resolution in localizing regional brain activity while BOLD fMRI has limited utility in tracking slow neural activity changes observed in fatigability studies (Aguirre et al., 1997, 2002).

Using magnetically labeled water in arterial blood as the diffusible tracer to provide quantification of regional cerebral blood flow (CBF), arterial spin labeling (ASL) perfusion fMRI is capable of non-invasively evaluating slow variations in neural activation patterns with high spatial and temporal resolution over long periods of time (Detre and Wang, 2002). ASL fMRI shows high stability over hours and days making it well suited to image fatigue and cognitive fatigability in PD, and it has been successfully used to assess brain function during both sustained cognitive tasks and task-free resting baselines (Wang et al., 2005; Kim et al., 2006; Olson et al., 2006; Rao et al., 2007a,b,c).

We have used ASL perfusion imaging and measured brain fatigue and time-on-task effects from sustained mental workload from continuous performance of the psychomotor vigilance test (PVT) in healthy individuals (Lim et al., 2010). The PVT is a simple reaction time task which provides a simple, reliable, and highly sensitive paradigm for capturing deficits in sustained attention and performance (Dorrian et al., 2005; Lim and Dinges, 2008). The attentional requirements in the PVT are free of aptitude or learning effects and are undiluted by elements of selectivity such as spatial orientation or executive decision-making, allowing it to be applied to all individuals and without adjustments for experience. These task features allowed us to isolate the effect of fatigability without having to account for potential confounding effects of differing visual stimuli, aptitude, strategy shifts or learning. Using the PVT in healthy subjects, we observed progressively slowing of reaction time (i.e., time-on-task effects) during task performance and increased mental fatigue ratings after the task (Lim et al., 2010). Moreover, regional CBF was significantly reduced in right fronto-parietal regions during post-task rest compared to pre-task rest, and the degree of regional CBF decrease correlated with the degree of performance decline, suggesting the key role of the fronto-parietal attention network in mediating brain fatigue from sustained mental workload.

In the present study, we employed the continuous 20-min PVT paradigm with ASL perfusion imaging to assess fatigue, cognitive fatigability, and brain activity changes in a cohort of PD patients. We aimed to examine whether PD patients with fatigue would (1) have difficulty completing the PVT due to motor deficits (van Rooden et al., 2009); (2) demonstrate cognitive fatigability and time-on-task effects in response to a sustained PVT task, and (3) show analogous neural changes as had been obtained in healthy adults (Lim et al., 2010).

## Materials and Methods

### Participants

A total of 21 PD patients (11 male, age range 52 to 80 years, mean age = 68 years) participated in this study. All participants were recruited from a large cohort of PD patients enrolled in the Udall Center at the University of Pennsylvania. PD participants had Montreal Cognitive Assessment (MoCA) score greater than 22, and reported normal sleep pattern (6–9 h range), as confirmed by the Pittsburgh Sleep Quality Index (PSQI) (Buysse et al., 1989). Patients were excluded if they had claustrophobia, depression, epilepsy, or other chronic debilitating medical conditions. Before the fMRI study, participants were required to have 7–8 h of sleep each night to minimize the potential effects of sleep debt on behavior and brain function. The Fatigue Severity Scale (FSS) was used to measure perceived general fatigue over recent period of time, day-to-day life. All subjects were compensated for participation in the study. Written consent was obtained according to the University of Pennsylvania Institutional Review Board.

### The PVT Task

The PVT (Dinges et al., 1997; Durmer and Dinges, 2005; Lim et al., 2010) was used as the sustained attention task in this study. This 20-min test is a simple reaction time test with varying and random inter-stimulus intervals (ISI) which range from 2 to 10 s (mean ISI = 6 s, including a 1s delay after each button press for subjects to read their reaction time). Participants were instructed to focus their attention on a red, rectangular box subtending 2 × 1.3 degrees of visual angle in the middle of a black screen. They were instructed to quickly stop the counter with a button press as soon as they saw a number displayed, but avoiding false starts. Immediately before and after administration of the PVT, patients rated their subjective mental fatigue score on the 9-point Visual Analog Scale (VAS). The following indexes were extracted as the measures of PVT performance, including mean reaction time (RT), median RT, standard deviation of RT, and number of lapses (defined as RT exceeding 500 ms). To assess time-on-task effects and performance fatigability, we divided the 20-min PVT into five 4-min quintiles and compared the median RT in the first quintile to that of the last quintile for each subject.

### Imaging Data Acquisition and Analyses

Functional Imaging data were acquired on a Siemens Magnetom 3T Prisma whole body scanner (Siemens AG, Erlangen, Germany), using a 64-channel head coil. All fMRI scans were conducted at the Hospital of the University of Pennsylvania. A spiral 3D pseudo-continuous arterial spin labeling (ASL) sequence was used for the perfusion scan with the following parameters: TR = 4 s, TE = 10 ms, flip angle = 90°, image matrix = 64 × 64, FOV = 240 mm, labeling time = 1.8 s, post labeling delay = 1.7 s. A total of 34 slices with 3.75 mm slice thickness were acquired in interleaved manner from anterior to posterior. High resolution T1-weighted structural images were acquired using a 3D MPRAGE sequence with the following parameters: TR = 2,400 ms, TE = 2.22 ms, flip angle = 8°, 208 slices with slice thickness of 0.80 mm, image matrix = 300 × 320, FOV = 256 mm. The baseline rest ASL protocol lasted for 6 min before and after PVT task and consisted of 36 acquisitions each. The PVT lasted for 20 min with 150 acquisitions.

Image data analysis was performed using Statistical Parametric Mapping (SPM 12) software (Wellcome Department of Cognitive Neurology, London), based in Matlab R2012b (Mathworks Inc., Natick, MA, United States). The ASL data processing was performed using fMRI Grocer toolbox^1^ and in house scripts (Dolui et al., 2019). Five subjects were excluded for imaging data analyses. Specifically, one subject was excluded due to missing VAS fatigue data. Two subjects were excluded due to large head movements greater than the size of a voxel (3.75 mm × 3.75 mm × 3.75 mm) and two other subjects were excluded due to coverage problems and CBF signal losing from ASL scans.

**FIGURE 1:**
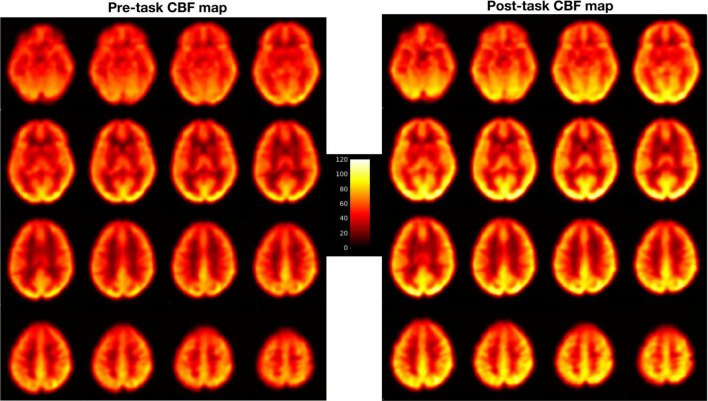
Pre- and post-task CBF maps of one representative PD patient.

Pre-processing steps included motion correction, coregistration, normalization and smoothing. Motion correction was done by aligning all the functional images to the mean image of the time series for each run to correct for the effects of head movements. Next, all the realigned images were coregistered to individual subject’s own T1 structural image. Then, perfusion weighted image series were generated by pair-wise subtraction of the label and control images (Wang et al., 2005). The resulting CBF-weighted images were averaged to obtain a mean CBF image for each condition. The mean CBF image was smoothed using a three-dimensional, 8 mm full width at half maximum Gaussian kernel and normalized to a 2 mm × 2 mm × 2 mm Montreal Neurological Institute (MNI) template. Similar to our previous study (Lim et al., 2010), the 20-min PVT data were divided into five 4-min quintiles, as follows: PVTq1 (the first quintile), PVTq2 (the second), PVTq3 (the third), PVTq4 (the fourth), and PVTq5 (the fifth). Each quintile resulted in its own smooth and warped mean CBF-weighted image. All the warped mean CBF maps were visually checked for optimal sensitivity, good contrast and intensity (see Figure 1 for an example of CBF maps). These mean CBF images were then entered into the whole brain voxel-wise general linear model (GLM) analysis. Contrasts were generated to compare the PVT with rest baselines (PVT vs Rest), the post-task resting baseline with the pre-task resting baseline (Rest2 vs Rest1), and the last quintile with the first quintile of PVT (PVTq5 vs PVTq1). Activation clusters were identified at a significance level of whole brain FWE corrected *p* < 0.05 with cluster size larger than 30 voxels.

In order to examine whether PD patients show analogous neural changes as had been obtained in healthy adults, region of Interest (ROI) analysis was also performed using *a priori* ROIs were defined from our previous study of fatigue and time-on-task effects in healthy subjects (Lim et al., 2010). These *a priori* ROIs included bilateral middle frontal gyrus (MFG), anterior cingulate cortex (ACC), superior temporal cortex (STC) and the right inferior parietal lobe (IPL). Regional CBF values were extracted for each ROI for both pre and post-task baselines. CBF changes (adjusting for global CBF difference) were calculated and correlated with performance changes and subjective fatigue measurements.

## Results

### Behavioral Data

**FIGURE 2:**
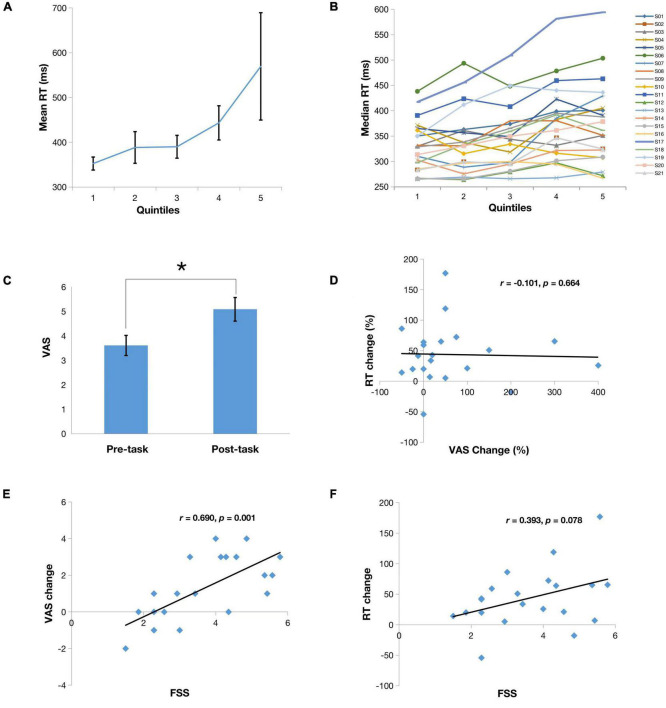
**(A)** Means of reaction time (RT) from the first to the last quintile. Note there is significant differences between mean RT in the last quintile and the first three quintiles (*p* < 0.05); **(B)** Median RT series of all subjects from the first to the last quintile; **(C)** Mean scores on a 9-point Visual Analog Scale (VAS) reported by subjects before and after the PVT. Note there is a significant mental fatigue increase after PVT compared to prior PVT (*p < 0.05); **(D)** Correlation of VAS change (%) to RT change (%); **(E)** Correlation of VAS change to Fatigue Severity Scale (FSS); **(F)** Correlation of RT change to FSS.

The median and mean RT for the PVT across all participants and all trials were 360.22 and 428.69 ms, respectively. On average subjects showed a steady increase in RT over 20 min of the PVT with median RT increasing from 331 to 389 ms and mean RT increasing from 352 to 569 ms (Figure 2A). Because there was less variance with median RT compared to mean, median RT was used to conduct subsequent analyses. One-way analysis of variance (repeated measures) of median RT over the five 4-min quintiles showed a significant effect of time (*F*_4,105_ = 2.67, *p* < 0.05). Similar to previous studies, we also observed large inter-individual differences. For instance, as shown in Figure 2B, subjects 1 and 17 showed a monotonic increase in RT, subjects 2, 4 and 5 appeared to reach plateau at 10 min but then continued to increase RT over the final 2 quintiles, and subjects 3 and 13 showed very little change in RT from the 1st quintile to the 5th quintile.

Patients also reported significantly increased mental fatigue rating on the after the 20-min PVT than before the PVT (Figures 2C, *p* < 0.05) and self-reported fatigue change significantly correlated with the FSS score (Figures 2E, *r* = 0.690, *p* = 0.001). These findings suggest that subjects with higher levels of perceived general fatigue have greater worsening of their fatigue while performing a sustained vigilance task. However, there was no correlation between RT change and self-reported mental fatigue change (Figure 2D, *r* = −0.101, *p* = 0.66), suggesting a dissociation between subjective fatigue and cognitive fatigability. In addition, there was a non-significant trend of correlation between RT change to the FSS score (Figure 2F, *r* = 0.393, *p* = 0.078).

### Imaging Results

**FIGURE 3:**
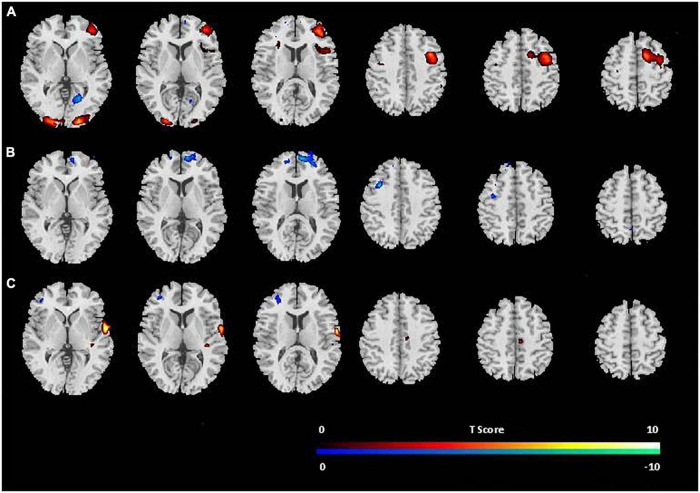
Brain areas with increase (red) or decrease (blue) in CBF **(A)** during PVT performance in comparison to the resting baselines; **(B)** post-task resting baseline in comparison to pre-task resting baseline; **(C)** during fifth quintile PVT scores in comparison to first quintile PVT scores. The threshold of display was set as uncorrected *p* < 0.001.

Figure 3 displays the results from whole brain analysis. When comparing the PVT to resting baselines, increased CBF was found in the right middle frontal gyrus (MFG), bilateral occipital cortex, and right cerebellum (see Figure 3A and Table 1A). When comparing post-task resting baseline to pre-task resting baseline, decreased CBF was found in the right MFG (BA 9, 10) while no increased CBF were observed (see Figure 3B and Table 1B). When comparing the first quintile of PVT to the last quintile of PVT, increased CBF was found in the right superior temporal cortex (STC; Figure 3C and Table 2).

**TABLE 1:** Brain areas showing **(A)** significant activation (CBF increases) and deactivation (CBF decreases) to the PVT compared to resting baselines (PVT vs. Rest) and **(B)** CBF decreases for post-task resting baseline compared to pre-task resting baseline (Rest 2 vs. Rest 1).

Region	Cluster size	MNI coordinates			Peak Z	Peak *p* (uncorrected)
		x	y	Z		
**(A) CBF increases: PVT vs. Rest**						
R. Middle frontal gyrus	976	32	40	20	5.55	< 0.001***
R. Occipital	386	20	−94	0	5.03	< 0.001*
R. Middle frontal gyrus	1218	36	8	42	4.73	< 0.001***
L. Occipital	384	−26	−94	4	4.65	< 0.001*
R. Cerebellum	310	40	−56	−22	3.74	< 0.001*
R. Inferior parietal gyrus	71	52	−40	34	3.88	< 0.001
R. Insula	126	48	16	14	3.64	< 0.001
**CBF decreases: PVT vs. Rest**						
R. Occipital	213	22	−60	−6	3.95	< 0.001
**(B) CBF decreases: Rest 2 vs. Rest 1**						
R. Middle frontal gyrus	317	10	58	18	4.03	< 0.001*
L. Middle frontal gyrus	59	−34	12	44	3.87	< 0.001

Thresholds were set as uncorrected p < 0.001. ***The clusters meet FWE corrected p < 0.001, *The clusters meet FWE corrected p < 0.05.L, left, R, right.

**TABLE 2:** Brain areas showing CBF changes for the last quintile of the PVT comparing to the first quintile of the PVT (PVTq5 vs. PVTq1).

Region	Cluster size	MNI coordinates			Peak Z	Peak *p*
		x	y	z		(uncorrected)
**CBF increases: PVTq5-PVTq1**						
R. Superior Temporal Gyrus	374	58	−2	0	3.89	< 0.001*
R. Hippocampus	57	34	−24	−6	3.43	< 0.001
**CBF decreases: PVTq1-PVTq5**						
L. Cerebellum	169	−2	−64	−22	3.80	< 0.001
L. Occipital Cortex	113	−50	−58	−6	3.54	< 0.001
L. Middle Frontal Gyrus	64	−30	26	30	3.53	< 0.001
L. Middle Frontal Gyrus	80	−30	40	16	3.42	< 0.001

Thresholds were set as uncorrected p < 0.001. *The clusters meet FWE corrected p < 0.05.L, left, R, right.

**FIGURE 4:**
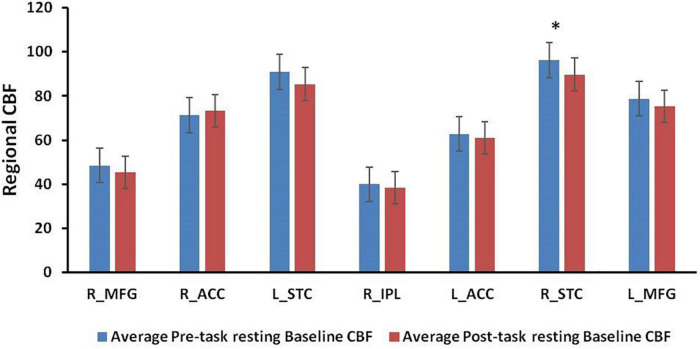
Regional CBF values at pre-task and post-task resting baselines in the region-of-interests (ROIs) from previous healthy control study. *p < 0.05.

**FIGURE 5:**
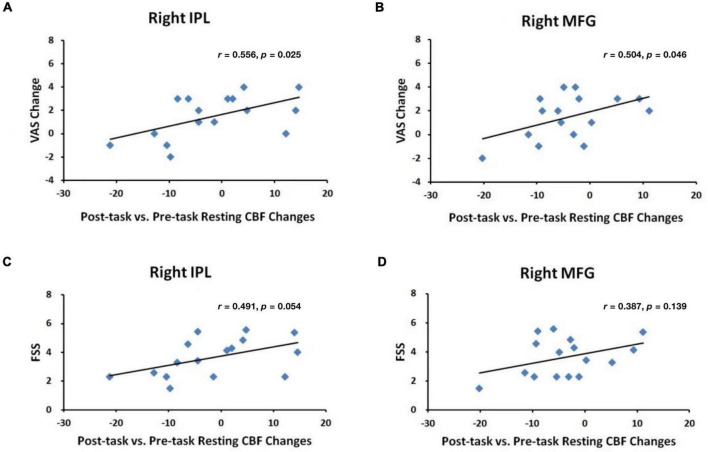
Correlations between VAS changes and post-task regional CBF changes in the **(A)** right IPL and **(B)** right MFG and between FSS scores and post-task regional CBF changes in the **(C)** right IPL and **(D)** right MFG.

Figure 4 displays the results from ROI analyses. Among the seven ROIs showing significantly reduced CBF in healthy adults (Lim et al., 2010), only the right STC (Figure 4, *p* = 0.030) showed a significant reduction in CBF between the pre-task and post-task baseline scans in PD patients. Regional CBF changes in the right IPL and the right MFG showed significant positive correlations with subjective fatigue (VAS) changes (IPL: *p* = 0.025, Figure 5A; MFG: *p* = 0.046, Figure 5B). There was also a trend of positive correlation between CBF changes in the right IPL and the FSS scores (*p* = 0.054, Figure 5C). There were no significant correlations between CBF changes in the right MFG and the FSS scores (*p* = 0.139, Figure 5D), and RT changes and regional CBF changes from pre-task to post-task baselines in any of the other ROIs (all *p* > 0.1).

## Discussion

This study evaluated fatigue and cognitive fatigability induced by performing a mentally challenging sustained attention task in a clinical sample of PD patients. As expected, PD patients in this study reported greater fatigue ratings after the task and displayed progressively increasing RT over the course of the PVT. These findings demonstrated that performance of a sustained attention task is associated with decrements in task performance and an increase in subjective fatigue in PD patients, suggesting that the research paradigms employed in this study can be used successfully to study induced fatigue in this population.

Previous research has suggested that PD patients might have difficulty completing the PVT due to motor deficits caused by the disease (van Rooden et al., 2009). However, our study demonstrated that, even though some PD patients had significant number of lapses, nearly all patients were able to complete the 20-min continuous PVT. Berard et al. (2020) also observed more lapses during the 20-min PVT in multiple sclerosis (MS), compared to healthy controls. It is possible that the 500 ms window, which was the lapse threshold defined by studying healthy volunteers, may need to be adjusted when studying clinical populations, who may have limiting motoric or sensory deficits. Notwithstanding this concern, our study suggests that the 20-min PVT is sufficient to invoke statistically significant differences in perceptions of fatigue and performance fatigability and supports the utility of continuous PVT as an appropriate paradigm to induce and examine fatigue in PD.

There were significant positive correlations of patients’ experience of fatigue in the week preceding the study (i.e., *via* the FSS) with their self-reported mental fatigue changes assessed immediately before and after the task (i.e., *via* a VAS), as well as a trend of correlation with performance declines during the PVT. However, mental fatigue changes did not correlate with task performance declines. These findings are consistent with a previous study reporting that PD-related fatigue is not associated with performance in a motor task without cue (Martino et al., 2016). However, they reported a link between PD-related fatigue and performance in an externally cued, attention-demanding version of the same task. These results are consistent with the notion that task-induced subjective fatigue and objective fatigability are two independent processes, though both appear to be influenced by perceived general fatigue before the task. That is, individuals with higher levels of general fatigue are more susceptible to the detrimental effects of sustained mental demands.

Significant task-related CBF changes were observed in the right MFG, bilateral occipital cortex, and the right cerebellum using ASL fMRI. The MFG activation in PD subjects is similar to the activation we found in healthy volunteers (Lim et al., 2010), indicating the critical role of the prefrontal cortex in tasks requiring vigilance and continuous performance in both the healthy and clinical populations. Similar activation of this area has also been reported in a variety BOLD fMRI studies employing comparable attention tasks (Fan et al., 2005; Ogg et al., 2008). The current study also found activation in cerebellum, which was not reported in our previous study. While primarily associated with motor control, the cerebellum has also been implicated in range of cognitive functions including attention (Brittain and Brown, 2013; Buckner, 2013). Thus, it is not surprising for the involvement of the cerebellum when performing the PVT in PD.

Compared to the pre-task resting baseline, post-task resting baseline scans showed significant deactivation in the right MFG, an area that overlapped with above PVT activated frontal region, indicating a persistent effect of brain fatigue in this area. Increased right MFG activation during the PVT is consistent with a previous study reporting compensatory hyper-activation in the fronto-parietal regions in PD patients to maintain attention performance and executive functions (Gerrits et al., 2015). The right MFG deactivation may reflect some residual cerebral effort in PD patients after a prolonged mental demanding task, which needs to be examined in future studies.

We also examined ROIs from our previous study where CBF decreases were observed from pre-task to post-task baselines in healthy subjects (Lim et al., 2010). In the current study, unlike healthy subjects, PD patients did not show significant CBF reduction from pre-task baseline to post-task baseline in most of these ROIs. Moreover, PD patients and healthy subjects showed differential correlations between changes in regional CBF, fatigue ratings, and PVT performance. In PD patients, regional CBF changes in the right MFG and the right IPL were significantly correlated with self-reported mental fatigue increases, but not RT increases. In contrast, regional CBF changes in these fronto-parietal regions were significantly correlated with RT increases, but not self-reported mental fatigue increases in healthy subjects (Lim et al., 2010). A potential explanation is that healthy subjects have the ability to consciously allocate their available cognitive and brain resources to preserve better performance over time, but such ability in PD patients may be impaired due to overload in their sustained attentional capacity during continuous time-on-task effects. As our findings demonstrated, the mean RT of the first quintile for PD patients is close to that of the last quintile in healthy subjects. The overloaded cognitive and brain activities may inevitably cause patients to experience fatigue. Taken together, these findings suggest that fronto-parietal regions may play different roles in mediating self-reported fatigue experience and cognitive fatigability in PD patients and healthy subjects, respectively.

Our study highlights the role of fronto-parietal attention network in mediating cognitive fatigue in PD, which are consistent with the fatigue literature. For example, Sepulcre et al. (2009) suggested an association between the symptom of fatigue and a disruption of brain networks involved in cognitive/attentional processes in MS. Our findings also suggest that using cerebral activity as a gauge for fatigability may result in improved correlations with subjective fatigue changes in persons with PD than performance declines.

The current study has several limitations. First, given the small sample size, our results may not be generalized to more heterogeneous samples. It is also unclear whether these findings are specific to PD patients. For example, fatigue in PD could be influenced by depressive symptoms (Katsarou et al., 2007). Even though those patients with major depressive disorder have been excluded form the present study, we cannot fully rule out the effect of depressive symptoms on the results. Thus future studies are needed to replicate these findings with a much larger sample size and better controlling for different confounding factors. Second, because no control group was included in this study, we were unable to compare the differential findings between PD patients and our previous study of healthy subjects (Lim et al., 2010). For example, CBF decrease from pre-task to post-task baseline scans was correlated with healthy individual’s ability to better maintain performance (Lim et al., 2010), while in the current study, we did not find such correlation in PD patients. Nevertheless, we speculate that this difference may be due to the minimal level of perceived general fatigue in healthy populations before the study. On the other hand, ages of PD patients in this study are much older than ages of healthy controls in our previous study (Lim et al., 2010), therefore we cannot be certain whether fatigue levels, demographic variables such as age, or their interaction ultimately explains the discrepancy. Future studies including both PD patients and age-matched healthy controls are necessary to understand how fatigue specifically manifests in the PD-affected brain under the same conditions as the healthy brain.

In summary, the current study utilized behavioral, psychological, and brain imaging measurements and demonstrated that the continuous PVT paradigm could successfully induce subjective fatigue and performance fatigability. We found that CBF changes in the right fronto-parietal network between the resting periods before and after the PVT were correlated with subjective fatigue changes, which suggest the critical role of this network in mediating fatigue in PD. These findings support the utility of continuous PVT paradigm to induce cognitive fatigue in PD and ASL perfusion imaging as a reproducible method for quantification of fatigue-related brain activity changes from sustained mental workload. Given that PD is a major global health concern affecting over 9 million people worldwide (Maserejian et al., 2020) and fatigue is one of the major non-motor symptoms of PD that impairs daily activity for PD patients, our study sheds light on the importance of understanding the brain mechanisms underlying fatigue to potentially help develop more effective treatment and prevention strategies.

## Data Availability Statement

The raw data supporting the conclusions of this article will be made available by the authors, without undue reservation.

## Ethics Statement

The studies involving human participants were reviewed and approved by the University of Pennsylvania Institutional Review Board. The patients/participants provided their written informed consent to participate in this study.

## Author Contributions

HR conceived the overall project and edited the manuscript. WL, RB, and EM conducted the study and analyzed the data. WL, JL, and RB drafted the original manuscript. All other authors reviewed and edited the manuscript for important scientific content and final approval.

## Conflict of Interest

The authors declare that the research was conducted in the absence of any commercial or financial relationships that could be construed as a potential conflict of interest.

## Publisher’s Note

All claims expressed in this article are solely those of the authors and do not necessarily represent those of their affiliated organizations, or those of the publisher, the editors and the reviewers. Any product that may be evaluated in this article, or claim that may be made by its manufacturer, is not guaranteed or endorsed by the publisher.

## Footnotes

1
https://www.nitrc.org/projects/fmri_grocer/
